# Global change of surgical and oncological clinical practice in urology during early COVID-19 pandemic

**DOI:** 10.1007/s00345-020-03333-6

**Published:** 2020-07-04

**Authors:** Robert Dotzauer, Katharina Böhm, Maximilian Peter Brandt, Peter Sparwasser, Maximilian Haack, Sebastian. Karl Frees, Mohamed Mostafa Kamal, René Mager, Wolfgang Jäger, Thomas Höfner, Igor Tsaur, Axel Haferkamp, Hendrik Borgmann

**Affiliations:** Department of Urology, University Medical Center, Johannes Gutenberg University, Langenbeckstr. 1, 55131 Mainz, Germany

**Keywords:** Coronavirus, SARS-CoV-2, Healthcare, Triage

## Abstract

**Objectives:**

While the coronavirus disease 2019 (COVID-19) pandemic captures healthcare resources worldwide, data on the impact of prioritization strategies in urology during pandemic are absent. We aimed to quantitatively assess the global change in surgical and oncological clinical practice in the early COVID-19 pandemic.

**Methods:**

In this cross-sectional observational study, we designed a 12-item online survey on the global effects of the COVID-19 pandemic on clinical practice in urology. Demographic survey data, change of clinical practice, current performance of procedures, and current commencement of treatment for 5 conditions in medical urological oncology were evaluated.

**Results:**

235 urologists from 44 countries responded. Out of them, 93% indicated a change of clinical practice due to COVID-19. In a 4-tiered surgery down-escalation scheme, 44% reported to make first cancellations, 23% secondary cancellations, 20% last cancellations and 13% emergency cases only. Oncological surgeries had low cancellation rates (%): transurethral resection of bladder tumor (27%), radical cystectomy (21–24%), nephroureterectomy (21%), radical nephrectomy (18%), and radical orchiectomy (8%). (Neo)adjuvant/palliative treatment is currently not started by more than half of the urologists. COVID-19 high-risk-countries had higher total cancellation rates for non-oncological procedures (78% vs. 68%, *p* = 0.01) and were performing oncological treatment for metastatic diseases at a lower rate (35% vs. 48%, *p* = 0.02).

**Conclusion:**

The COVID-19 pandemic has affected clinical practice of 93% of urologists worldwide. The impact of implementing surgical prioritization protocols with moderate cancellation rates for oncological surgeries and delay or reduction in (neo)adjuvant/palliative treatment will have to be evaluated after the pandemic.

**Electronic supplementary material:**

The online version of this article (10.1007/s00345-020-03333-6) contains supplementary material, which is available to authorized users.

## Introduction

The coronavirus disease 2019 (COVID-19) outbreak has led to significant medical, political, economic and socio-cultural changes. Declared as a pandemic on March 11, 2020 by the World Health Organization (WHO), COVID-19 is globally threatening the human population, with currently 4.262.799 infections, 291,981 deaths in 185 countries at May 13, 2020 [1, 2]. Different strategic actions have been taken to reduce the rate of new infections like isolation of infected patients and their contacts, mass quarantine, school and university closures and even curfews [3]. At the current stage of the pandemic, health care systems all over the world are rearranging human and material resources to provide increasing capacities for patients with COVID-19 [4]. Currently, clinicians worldwide have to deal with a lack of intensive care capacities and its ethical uncertainties as it has been known in smaller extent from pandemic influenza [5].

Not only because of fading intensive care resources as backup for major surgical procedures but also to withhold staff capacities for potential involvement in the treatment of COVID-19 patients, surgical departments have to evaluate down-escalation and prioritization of surgeries [6]. These prioritization plans to suspend elective surgery can lead to serious ethical questions, since delay of patients surgery might result in short-term clinical progression of cancer and increased cancer specific mortality [7]. In bladder cancer, for example, prolonged time to cystectomy > 3 months was associated with adverse disease-specific and overall survival in two studies after diagnosis and neoadjuvant chemotherapy [8, 9]. Moreover, in medical oncology, disease spread and cancer related mortality must be weighted for palliative and (neo)adjuvant treatments against the patient’s risk of a COVID-19 infection and its required healthcare resources [10]. Robust scientific data on prioritization strategies in surgery and decision-making in medical oncology during pandemic are currently absent.

Therefore, we aimed to quantitatively assess the global change in surgical and oncological clinical practice in Urology during COVID19 pandemic. This cross-sectional global overview might serve clinicians as benchmark for their current management and as forecast on upcoming changes in the surgical and medical treatment of their patients.

## Methods

For this cross-sectional global study, we used the platform www.surveymonkey.com and designed a 12-item online survey (see Supplementary data) on the global effects of the COVID-19 pandemic on the surgical and oncological management of urology departments. The survey was designed in accordance with the Checklist of Reporting Results of internet-E-Surveys (CHERRIES) [11]. For successful completion of the survey, answering all questions was not mandatory. All members of our working group piloted the survey. No technical problems occurred, but we modified certain wording to improve understanding. Finally, the survey showed high face validity and was tested with 5 volunteers.

The target population consisted of urologic oncologists and urologic surgeons worldwide. This was queried by the first question. Answering “No” would result in ending the survey. The survey assessed the participant’s country of work and its geographical risk-status (high-risk for COVID-19; yes vs. no vs. no answer), type of the participant’s hospital (private vs. public vs. other), change of the clinical practice because of COVID-19 pandemic (yes vs. no) and current performance (yes vs no) of 13 oncological and 4 non-oncological common surgical procedures (author group consensus). Moreover, the hospital’s stage of down escalation of surgical activity based on the “Considerations in the triage of urologic surgeries during the COVID-19 pandemic” [6], the participant’s country of work, a COVID-19 training of the medical staff (yes vs. no), an involvement of the staff in the specific treatment of COVID-19-infections (yes vs. no), the occurrence of COVID-19 infections in the participant’s hospital (patient in the hospital and/or patient in the department and/or healthcare professional in the hospital and/or healthcare professional in the department vs. none of the above) and the date when clinical practice changed (day/month/year) were evaluated. Concerning medical urological oncology, participant’s wear asked if (neo)adjuvant/palliative treatment is currently started (yes/no) for 5 clinical conditions which had been described as worthwhile for reconsideration during the current COVID-19 pandemic [10].

On 19 MAR 2020, the survey was disseminated in a non-targeted fashion in Social Media on Twitter (using the hashtags #COVID19, #Urology, #Survey, and #Coronavirus) and on Facebook (1 post each in 10 Facebook urology groups). We chose to perform data cut-off on 24 MAR 2020, 100 h after first dissemination of the survey. The study was performed in accordance with WHO’s Guidance for managing ethical issues in infectious disease outbreaks [12]. Methodology adhered to the Strengthening the Reporting of Observational Studies in Epidemiology (STROBE) statement [13].

Statistical analysis was performed using IBM SPSS Statistics Version 20 (Armonk, NY: IBM Corp.). Descriptive statistics were reported as frequencies and proportions. Mean values were calculated for countries’ stage of surgery down escalation in case of multiple responses per country. Countries’ performance of (non)oncological surgical procedures and oncological systemic treatments according to geographical COVID-19 risk status (high-risk vs. non-high-risk) was compared using Chi-squared test. Significance level was set to *p* = 0.05.

## Results

At data cut-off on March 24 2020, we received a total of 260 survey responds, of which 25 (9%) were not urologists (survey question one) and thus immediate drop-outs. A total of 235 urologists from 44 different countries covering all 6 continents responded to the survey. From these, 131 (63%) were practicing in high-risk countries, 57 (27%) in non-high-risk countries and 21 (10%) stated no answer. 43 (20%) were working in private hospitals, 160 (76%) in public hospitals and 8 (4%) stated “other”. Supplementary Tables 1 and 2 list the number of respondents from each country as well as the number of survey responses to each question.

**Fig. 1:**
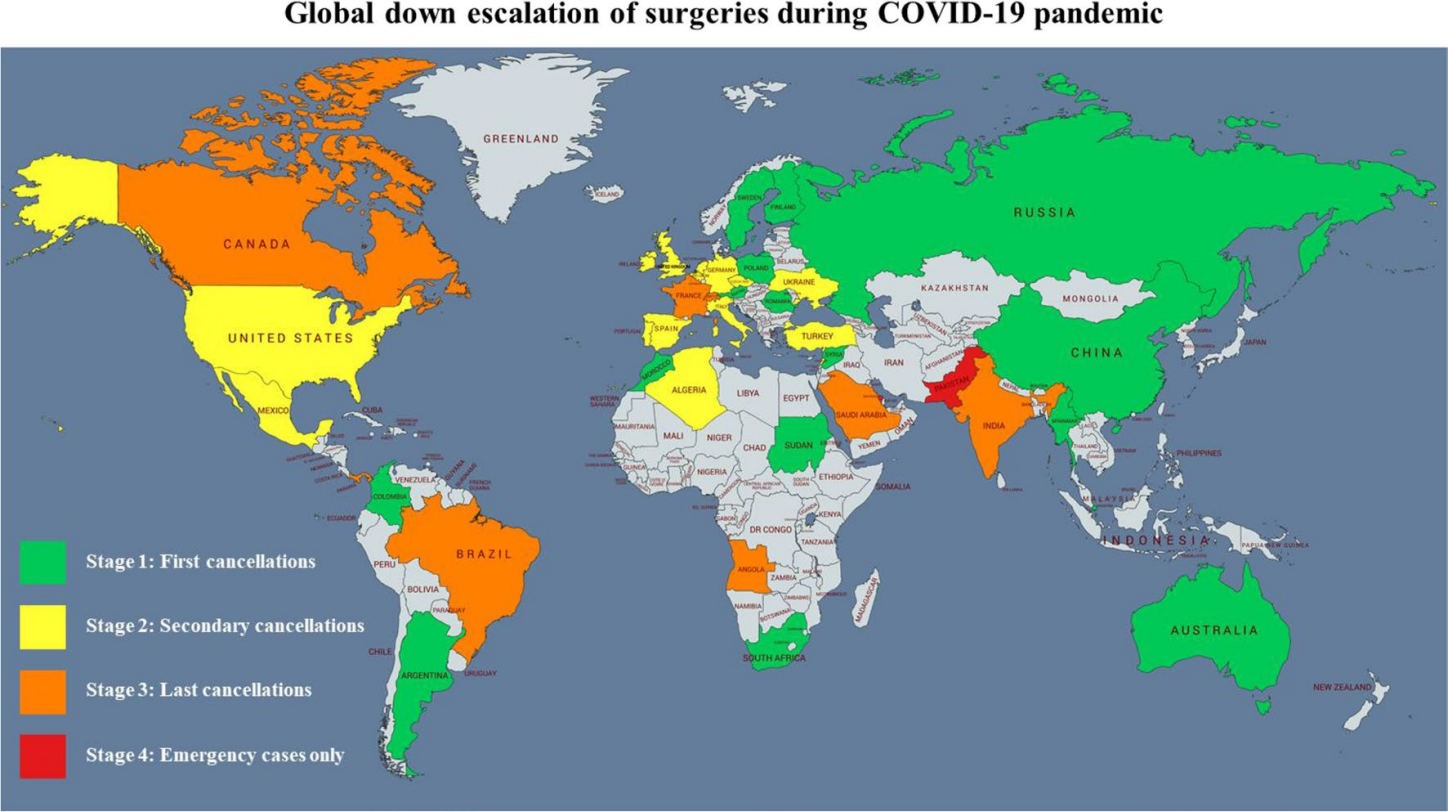
World map picturing survey responds on four stages of down escalation activity in surgical management in urology

The vast majority of 93% of urologists indicated a change of clinical practice due to COVID-19-pandemic, 7% indicated no change of clinical practice. Concerning surgery cancellations, step-wise down-escalation activity is demonstrated in Fig. 1. 77 (44%) urologists reported to be in stage 1 (First cancellations), 40 (23%) in stage 2 (Secondary cancellations), 35 (20%) in stage 3 (Last cancellations) and 23 (13%) in stage 4 (Emergency cases only).

**Fig. 2:**
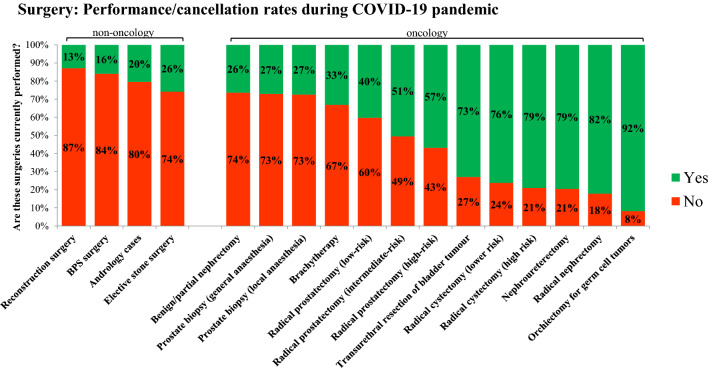
Surgery: current performance/cancellation rates for 4 non-oncological and 13 oncological procedures during COVID-19 pandemic by 235 urologists worldwide

Figure 2 highlights the urologists’ prioritization of surgeries for oncological and non-oncological operations. Surgeries with highest cancellation rates (> 70%) were: reconstruction surgery (87%), surgery for benign prostate syndrome (84%), andrology cases (80%), elective stone surgery (74%), benign/partial nephrectomy (74%) and prostate biopsy (73%). Surgeries with low cancellation rates (< 30%) were: transurethral resection of bladder tumor (27%), radical cystectomy (21-24%), nephroureterectomy (21%), radical nephrectomy (18%), radical orchiectomy (8%).

**Fig. 3:**
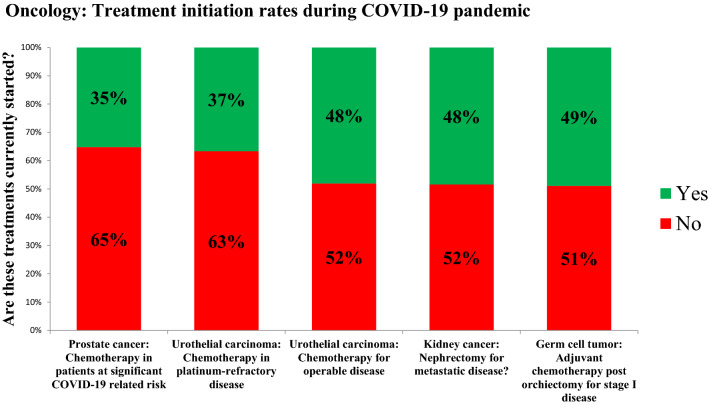
Oncology: current treatment initiation rates of (neo)adjuvant/palliative treatment for 5 clinical conditions in metastatic/high-risk urologic cancer

Concerning medical urological oncology, Fig. 3 shows that (neo)adjuvant/palliative treatment is currently not started by more than half of the urologists in 5 specific clinical conditions for prostate cancer (65%), urothelial carcinoma (recurrent disease, 63%), urothelial carcinoma (neoadjuvant, 63%), kidney cancer (52%) and testicular cancer (52%).

Less than half of respondents (*n* = 74, 42%) received a specific COVID-19 training for their staff. In 30 cases (17%) the urologic staff was already involved in the specific treatment of patients for COVID-19. 124 (72%) respondents indicated a confirmed COVID-19 patient in their hospital and 18 (10%) in their department. 20 (12%) respondents stated a COVID-19 infection of a health care professional in their department and 72 (42%) in their hospital. In 38 (22%) cases no COVID-19 infection was reported. Median date when clinical practice changed was 16 March 2020 (interquartile range 13 March 2020–18 March 2020).

In a between-countries risk-stratified comparison, COVID-19 high-risk-countries had higher total cancellation rates (78%) for non-oncological procedures than non-high-risk-countries (68%, *p* = 0.01). Moreover, less oncological treatments for metastatic diseases were started in high-risk countries (cumulative rate for treatment start: 35%) than in non-high-risk countries (cumulative rate for treatment start: 48%, *p* = 0.02). Total cancellation rates for oncological procedures were comparable between high-risk (44%) and non-high-risk countries (45%, *p* = 0.68).

## Discussion

In a worldwide disseminated online survey with 260 respondents, we assessed the latest global effects of COVID-19 pandemic on surgical and oncological clinical practice in urology. A dramatic change of daily clinical practice in surgical and medical treatment of urologic patients was observed.

Regarding global changes in surgical management, the current level of escalating down activity (world map, Fig. 1) reflects the different strategies of health care systems with their diverse abilities to prevent, identify, and respond to the COVID-19 outbreak [14]. Interestingly, cancellation rates of oncological surgeries did not differ between high-risk and non-high-risk countries whereas high-risk countries had more cancellations of non-oncological surgeries. Oncological surgery for urothelial carcinoma, kidney cancer and testicular cancer was still performed by the majority of respondents probably due to the risk for disease progression and association of delayed surgery with impaired surgical conditions and adverse survival outcomes [15–18].

Interpreting our survey data on performance/cancellation rates of urological surgery (Fig. 2), it must be kept in mind that certain hospitals might not perform certain operations (e.g. cystectomy) at baseline during non-pandemic times. In this concern, the COVIDSurg Collaborative has most recently reported best estimates for cancellation rates of surgery based on a global expert-response study and using a Bayesian beta-regression model. In urology, global elective surgery cancellations are estimated to be as high as 458,151 (cancellation rate 36.6%) for cancer surgery and 2,492,604 for benign surgery (cancellation rate: 81.7%) [19].

As delay of surgery for urothelial carcinoma is associated with adverse survival outcome, it is highly remarkable that 21% of urologists are currently not performing radical cystectomy for high-risk cancer. This could be explained by lack of intensive care units for routine early postoperative care but also by shortage of medical staff. In order to further prioritize oncological surgery, various stratifications of urological surgery have been proposed, like the five categories from the Cleveland Clinic Department of Urology: (0) Emergency (obstructed kidney/infection, torsion etc.); (1) cystectomy—high risk cancer, orchiectomy, etc. (2) radical prostatectomy—high risk cancer, radical nephrectomy; etc. (3) cystectomy—not high risk cancer, partial nephrectomy, etc. (4) nonessential (radical prostatectomy for low risk cancer, partial nephrectomy for small renal masses, etc. [20]. Triage of uro-oncological surgeries, however, leads to an increasing burden of delayed surgeries which will absorb medical working capacity in the near future [21]. Moreover, the burden of non-oncological therapies in functional urology and stone disease is accumulating [22, 23], calling for novel strategies not to compromise the quality of life of patients. In this regard, the field of urology has the chance not only to adapt to the current pandemic by taking immediate actions for outpatient clinics, operating rooms, department structure and research, but also to prepare for its intermediate and long-term future by integrating telemedicine and technology into routine clinical practice [24].

Regarding initiation of treatment for metastatic disease, half of the respondents (52%) stated not to perform radical nephrectomy currently if metastases are present. Since there is valid data for medical systemic treatment alone for metastatic renal-cell carcinoma, surgical capacities and the potential need for intensive care units might be restrained [25]. Two-thirds (65%) of the respondents answered not to start a chemotherapy in prostate cancer patients at significant COVID-19-related risk. Neutropenia as one of the major side effects of chemotherapy leading to impaired immune response and availability of modern androgen receptor targeted agents might be factors leading to this decision [26, 27]. High-risk countries reported to start fewer treatments for metastatic disease than non-high-risk countries. The implications of the COVID-19 pandemic on cancer patients due to limited diagnostic and treatment capacities are unknown yet, negative effects on long-term outcomes should be anticipated [28].

At this moment, the risk assessment for cancer patients of disease progression due to a delay of an oncologic therapy versus acquiring a COVID-19 infection during a (neo)adjuvant/palliative therapy remains an individual physician’s decision. Meanwhile, prioritization and screening protocols concerning cancer treatment during COVID-19 pandemic are being introduced like in The Seattle Cancer Care Alliance (WA, USA) [29]. In this line, the official French guidelines to protect patients with cancer against SARS-CoV-2 infection have been proposed [30]. These include a prioritization order integrating the essence of intent therapeutic strategy (curative or non-curative), age, life expectancy, time since diagnosis and symptoms.

Our study has several limitations. Overall, due to the survey methodology, answers were self-reported, not mandatory for all questions and not objectively assessed. Regional differences within a country are likely to be present leading to an inherent bias in survey responds. Moreover, being dependent on established urology specific dissemination strategies on twitter for capturing a global picture of the effects of the COVID-19 pandemic in a highly dynamic situation, only urologist (surgical and oncological) participated in the survey. Furthermore, due to the urgency of the topic resulting in a short time period of only 100 h of acquisition, the number of respondents is limited. Finally, survey response rate could not be calculated due to social media dissemination with untraceable number of message recipients.

Despite these shortcomings, our study provides the first global picture of a dramatic change in surgical and oncological clinical practice due to the COVID-19 pandemic. Our cross-sectional global data might serve as a kick-off for clinical and epidemiological studies investigating the effects of crisis management on clinically-relevant outcomes. At this moment, it serves clinicians as benchmark for their current management and as forecast on upcoming changes in the surgical and medical treatment of their patients.

## Conclusion

The COVID-19 outbreak has affected clinical practice of 93% of urologists worldwide. This led to surgery prioritization with moderate cancellation rates for oncological surgeries and to re-consideration of commencing (neo)adjuvant/palliative treatment in certain metastatic disease stages. Clinical and epidemiological studies are needed investigating survival and quality of life outcomes to provide guidelines for surgical and medical oncological clinical practice in case of a global state of crisis.

## Electronic supplementary material

Below is the link to the electronic supplementary material.

Supplementary material 1 (DOCX 16 kb)

Supplementary material 2 (DOCX 17 kb)

## References

[1] Jones DS (2020). History in a crisis—lessons for Covid-19. N Engl J Med.

[2] John-Hopkins University (2020) Coronavirus Resource Center. From: https://coronavirus.jhu.edu. Accessed 13 May 2020

[3] Niud Y, Xu F (2020). Deciphering the power of isolation in controlling COVID-19 outbreaks. Lancet Glob Health.

[4] Rosenbaum L (2020). Facing Covid-19 in Italy—ethics, logistics, and therapeutics on the epidemic’s front line. N Engl J Med.

[5] Silva DS, Gibson JL, Robertson A (2012). Priority setting of ICU resources in an influenza pandemic: a qualitative study of the Canadian public’s perspectives. BMC Public Health.

[6] Stensland KD, Morgan TM, Moinzadeh A (2020). Considerations in the triage of urologic surgeries during the COVID-19 pandemic. Eur Urol.

[7] Bleicher RJ, Ruth K, Sigurdson ER (2016). Time to surgery and breast cancer survival in the United States. JAMA Oncol.

[8] Lee CT, Madii R, Daignault S (2006). Cystectomy delay more than 3 months from initial bladder cancer diagnosis results in decreased disease specific and overall survival. J Urol.

[9] Boeri L, Soligo M, Frank I (2019). Delaying Radical Cystectomy After Neoadjuvant Chemotherapy for Muscle-invasive Bladder Cancer is Associated with Adverse Survival Outcomes. Eur Urol Oncol.

[10] Gillessen S, Powles T (2020). Advice Regarding Systemic Therapy in Patients with Urological Cancers During the COVID-19 Pandemic. Eur Urol.

[11] Eysenbach G (2004). Improving the quality of Web surveys: the Checklist for Reporting Results of Internet E-Surveys (CHERRIES). J Med Internet Res.

[12] World Health Organization (2020) Ethical standards for research during public health emergencies: distilling existing guidance to support COVID-19 R&D. https://apps.who.int/iris/handle/10665/331507. Accessed 11 May 2020

[13] von Elm E, Altman DG, Egger M (2007). The Strengthening the Reporting of Observational Studies in Epidemiology (STROBE) statement: guidelines for reporting observational studies. Lancet.

[14] Kandel N, Chungong S, Omaar A, Xing J (2020). Health security capacities in the context of COVID-19 outbreak: an analysis of International Health Regulations annual report data from 182 countries. Lancet.

[15] Froehner M, Heberling U, Zastrow S (2016). Growth of a level iii vena cava tumor thrombus within 1 month. Urology.

[16] Bourgade V, Drouin SJ, Yates DR (2014). Impact of the length of time between diagnosis and surgical removal of urologic neoplasms on survival. World J Urol.

[17] Chan VW, Chiu PK, Yee CH, Yuan Y, Ng CF, Teoh JY (2020) A systematic review on COVID-19: urological manifestations, viral RNA detection and special considerations in urological conditions. World J Urol. 10.1007/s00345-020-03246-4 10.1007/s00345-020-03246-4 PMC7251800 32462305

[18] Zehnder P, Thalmann GN (2013). Timing and outcomes for radical cystectomy in nonmuscle invasive bladder cancer. Curr Opin Urol.

[19] COVIDSurg Collaborative (2020) Elective surgery cancellations due to the COVID-19 pandemic: global predictive modelling to inform surgical recovery plans. Br J Surg. 10.1002/bjs.11746 10.1002/bjs.11746 PMC7272903 32395848

[20] Goldman HB, Haber GP (2020) Recommendations for tiered stratification of urological surgery urgency in the COVID-19 Era. J Urol 204(1):11–3. 10.1097/JU.0000000000001067 10.1097/JU.0000000000001067 PMC7273865 32249715

[21] Oderda M, Roupret M, Marra G et al (2020) The impact of COVID-19 outbreak on uro-oncological practice across Europe: which burden of activity are we facing ahead? Eur Urol 78(1):124–126. 10.1016/j.eururo.2020.04.036 10.1016/j.eururo.2020.04.036 PMC7180374 32349934

[22] Proietti S, Gaboardi F, Giusti G (2020) Endourological Stone Management in the Era of the COVID-19. Eur Urol. 10.1016/j.eururo.2020.03.042 10.1016/j.eururo.2020.03.042 PMC7195508 32303384

[23] Phe V, Karsenty G, Robert G et al (2020) Widespread postponement of functional urology cases during the COVID-19 pandemic: rationale, potential pitfalls, and future consequences. Eur Urol 78(1):4–5. 10.1016/j.eururo.2020.04.031 10.1016/j.eururo.2020.04.031 PMC7177126 32349933

[24] Margel D, Ber Y (2020) Changes in urology after the first wave of the COVID-19 pandemic. Eur Urol Focus 10.1016/j.euf.2020.05.001 10.1016/j.euf.2020.05.001 PMC7218385 32405544

[25] Mejean A, Ravaud A, Thezenas S (2018). Sunitinib alone or after nephrectomy in metastatic renal-cell carcinoma. N Engl J Med.

[26] Sweeney CJ, Chen YH, Carducci M (2015). Chemohormonal therapy in metastatic hormone-sensitive prostate cancer. N Engl J Med.

[27] Davis ID, Martin AJ, Stockler MR (2019). Enzalutamide with standard first-line therapy in metastatic prostate cancer. N Engl J Med.

[28] Naspro R, Da Pozzo LF (2020). Urology in the time of corona. Nat Rev Urol.

[29] Burki TK (2020) Cancer care in the time of COVID-19. Lancet Oncol 21(5):628. 10.1016/S1470-2045(20)30201-1 10.1016/S1470-2045(20)30201-1 PMC7156149 32213339

[30] You B, Ravaud A, Canivet A (2020). The official French guidelines to protect patients with cancer against SARS-CoV-2 infection. Lancet Oncol.

