# Supplementary material for: Global change of surgical and oncological clinical practice in urology during early COVID-19 pandemic
Source: World J Urol. 2020 Jul 4;39(9):3139–45. doi: 10.1007/s00345-020-03333-6 (PMC7335229; doi:10.1007/s00345-020-03333-6)
Supplement: Supplementary file 1 — Supplementary material 1 (DOCX 16 kb) [file 345_2020_3333_MOESM1_ESM.docx]

**Supplementary Table 1.** Number of survey responds per country.

| Country | Number of responds (% of all responds) |
| --- | --- |
| United States of America | 25 (9.61%) |
| Netherlands | 24 (9.23%) |
| Germany | 23 (8.84%) |
| Spain | 14 (5.38%) |
| United Kingdom of Great Britain and Northern Ireland | 12 (4.61%) |
| France | 10 (5.43%) |
| Belgium | 9 (3.46%) |
| Turkey | 9 (3.46%) |
| Italy | 7 (2.69%) |
| Canada | 3 (1.15%) |
| India | 3 (1.15%) |
| Ukraine | 3 (1.15%) |
| Argentina | 2 (0.77%) |
| Brazil | 2 (0.77%) |
| Czech Republic | 2 (0.77%) |
| Ireland | 2 (0.77%) |
| Mexico | 2 (0.77%) |
| Morocco | 2 (0.77%) |
| Poland | 2 (0.77%) |
| Romania | 2 (0.77%) |
| Russian Federation | 2 (0.77%) |
| Switzerland | 2 (0.77%) |
| Afghanistan | 1 (0.38%) |
| Algeria | 1 (0.38%) |
| Angola | 1 (0.38%) |
| Australia | 1 (0.38%) |
| Austria | 1 (0.38%) |
| Bahrain | 1 (0.38%) |
| Chile | 1 (0.38%) |
| China | 1 (0.38%) |
| Colombia | 1 (0.38%) |
| Finland | 1 (0.38%) |
| Jamaica | 1 (0.38%) |
| Lebanon | 1 (0.38%) |
| Myanmar | 1 (0.38%) |
| Pakistan | 1 (0.38%) |
| Panama | 1 (0.38%) |
| Portugal | 1 (0.38%) |
| Saudi Arabia | 1 (0.38%) |
| Singapore | 1 (0.38%) |
| South Africa | 1 (0.38%) |
| Sudan | 1 (0.38%) |
| Sweden | 1 (0.38%) |
| Syrian Arab Republic | 1 (0.38%) |
