# Supplementary material for: Global change of surgical and oncological clinical practice in urology during early COVID-19 pandemic
Source: World J Urol. 2020 Jul 4;39(9):3139–45. doi: 10.1007/s00345-020-03333-6 (PMC7335229; doi:10.1007/s00345-020-03333-6)
Supplement: Supplementary file 2 — Supplementary material 2 (DOCX 17 kb) [file 345_2020_3333_MOESM2_ESM.docx]

**Supplementary Table 2.** Number of survey responds to each survey question.

| Question Number | Question Content | Answers | Skipped |
| --- | --- | --- | --- |
| 1 | Are you a Urologist? | 260 | 0 |
| 2 | Is your country of work a WHO high-risk country? | 209 | 51 |
| 3 | In which type of hospital are you working? | 211 | 49 |
| 4 | Has your clinical practice changed because of COVID-19? | 211 | 49 |
| 5 | Urological Surgery: Are the following operations currently performed at your hospital? | 188 | 72 |
| 6 | Imagine four stages of escalate down activity: In which stage are you at this moment? - Please choose the most applicable answer.   - Stage 1: First cancellations (BPS surgeries, vasectomies, circumcisions, prostate biopsies [low PSA], benign/partial nephrectomies, radical prostatectomies [low-risk], elective URS / PCNL) - Stage 2: Secondary cancellations (Cystectomies [lower risk cancer], prostate biopsies [high PSA], TURBT [low risk cancer], radical prostatectomies [high-risk], nephroureterectomies) - Stage 3:  Last to be cancelled (Cystectomies [high risk cancer], TURBT [high risk cancer], nephrectomies, orchiectomies) - Stage 4: Emergency cases only (Obstructed kidneys/infection, abcesses, torsions) | 175 | 85 |
| 7 | Country of work: | 184 | 76 |
| 8 | Has there been a specific COVID-19 training for your staff? | 173 | 87 |
| 9 | Is your staff involved in the specific treatment of COVID-19-Infections? | 173 | 87 |
| 10 | There has been a positive COVID-19 test (infection proved) for a   - Patient in our hospital? - Patient in our department? - Health-care-professional in our hospital? - Health-care-professional in our department? - None of the above | 173 | 87 |
| 11 | When has your clinical practice changed? | 168 | 92 |
| 12 | Urological Oncology: Are these treatments currently started? | 161 | 99 |
| 13 | Please feel free to leave any comments: | 22 | 238 |
